# Early reduction of pulmonary arterial hypertension in patients using a long-term mechanical ventricular assistance device: a cross-sectional study

**DOI:** 10.1590/1516-3180.2021.0944.R2.18032022

**Published:** 2022-05-05

**Authors:** Bruno Soares da Silva Rangel, Bruno Biselli, Nádia Romanelli Quintanilha, Mônica Samuel Avila, Paulo Manuel Pêgo-Fernandes, Fabio Biscegli Jatene, Roberto Kalil, Silvia Moreira Ayub Ferreira

**Affiliations:** I MD. Attending Physician, Hospital Sírio-Libânes (HSL), São Paulo (SP), Brazil; and Attending Physician, Hospital Vila Nova Star, São Paulo (SP), Brazil.; II MD. Attending Physician, Instituto do Coração, Hospital das Clínicas HCFMUSP, Faculdade de Medicina, Universidade de São Paulo, São Paulo (SP), Brazil; and Attending Physician, Hospital Sírio-Libânes (HSL), São Paulo (SP), Brazil.; III MD. Attending Physician, Instituto do Coração, Hospital das Clínicas HCFMUSP, Faculdade de Medicina, Universidade de São Paulo, São Paulo (SP), Brazil.; IV MD, PhD. Attending Physician, Instituto do Coração, Hospital das Clínicas HCFMUSP, Faculdade de Medicina, Universidade de São Paulo, São Paulo (SP), Brazil; and Attending Physician, Hospital Sírio-Libânes (HSL), São Paulo (SP), Brazil.; V MD, PhD. Full Professor, Thoracic Surgery Program, Instituto do Coração, Hospital das Clínicas HCFMUSP, Faculdade de Medicina, Universidade de São Paulo, São Paulo (SP), Brazil; and Cardiothoracic Surgeon, Hospital Sírio-Libânes (HSL), São Paulo (SP), Brazil.; VI MD, PhD. Full Professor, Cardiovascular Surgery Division, Instituto do Coracao, Hospital das Clinicas HCFMUSP, Faculdade de Medicina, Universidade de Sao Paulo, Sao Paulo, SP, BR; and Cardiovascular Surgeon, Hospital Sírio-Libanês (HSL), São Paulo (SP), Brazil.; VII MD, PhD. Full Professor, Cardiology Division, Instituto do Coracao, Hospital das Clinicas HCFMUSP, Faculdade de Medicina, Universidade de Sao Paulo, Sao Paulo, SP, BR; and General Director, Cardiology Center, Hospital Sírio-Libanês (HSL), São Paulo (SP), Brazil.; VIII MD, PhD. Attending Physician, Instituto do Coração, Hospital das Clínicas HCFMUSP, Faculdade de Medicina, Universidade de São Paulo, São Paulo (SP), Brazil; and Attending Physician, Hospital Sírio-Libânes (HSL), São Paulo (SP), Brazil.

**Keywords:** Pulmonary arterial hypertension, Heart failure, Heart-assist devices, Ventricular assistance device, Pulmonary arterial hypertension, Heart failure, Heart transplant

## Abstract

**BACKGROUND:**

Severe pulmonary arterial hypertension (PAH) is a contraindication for heart transplantation (HT). It has been correlated with increased early and late mortality, mainly associated with right ventricular failure. Ventricular assistance devices (VADs) can promote reduction of intracardiac pressures and consequent reduction of PAH over the medium and long terms, thus enabling future candidature for HT. The diminution of early pulmonary pressure within this scenario remains unclear.

**OBJECTIVE:**

To evaluate the reduction of PAH and correlate data from right catheterization with the earliness of this reduction.

**DESIGN AND SETTING:**

Cross-sectional study in a general hospital in São Paulo, Brazil.

**METHODS:**

This was a retrospective analysis on the medical records of patients undergoing VAD implantation in a single hospital. Patients for whom VAD had been indicated as a bridge to candidature for HT due to their condition of constant PAH were selected.

**RESULTS:**

Four patients with VADs had constantly severe PAH. Their mean pulmonary artery systolic pressure (PASP) before VAD implantation was 66 mmHg. Over the 30-day period after the procedure, all the patients evolved with a drop in PASP to below 60 mmHg. Their new average was 36 mmHg, which was a drop of close to 50% from baseline values. The one-year survival of this sample was 100%.

**CONCLUSION:**

VAD implantation can reduce PAH levels. Early reduction occurred in all patients. Thus, use of VAD is an important bridge tool for enabling candidature for HT among patients with constantly severe PAH.

## INTRODUCTION

Despite advances in the therapeutic arsenal for treating heart failure, comprising lifestyle changes, pharmacological therapy and cardiac stimulation devices, a considerable portion of the population progresses to refractoriness. In such situations, more advanced treatments such as heart transplantation or use of a ventricular assistance device (VAD) need to be considered. Heart transplantation remains the therapy of choice and provides greater life expectancy for patients with advanced heart failure.^1^ However, technological advances involving use of VADs, with reduction of adverse events and greater management experience among specialists, have allowed greater availability, use and tolerability of this method worldwide, including increasing use of this therapy in Brazil. VADs can be used as a bridge to transplantation or as the destination therapy.^2^

Constant pulmonary arterial hypertension (PAH) is one of the main contraindications for heart transplantation.^1,3^ In this context, it has been observed, after VAD implantation, that patients with contraindications for heart transplantation due to pulmonary arterial hypertension presented decompression of the left cardiac cavities and reverse remodeling of the pulmonary vessels, with reversal of PAH. This gave rise to the possibility of candidature for heart transplantation. Data in the current literature show that this change occurs over the first three to six months after VAD implantation,^1^ with some reports of earlier reduction.

## OBJECTIVE

The objectives of the present study were to evaluate the reduction of PAH and correlate data from right catheterization with the earliness of this reduction.

## METHODS

This was a retrospective evaluation on patients with advanced heart failure who underwent VAD implantation between 2013 and 2019, through data collection from the electronic medical records. Patients for whom VAD had been indicated as a bridge to candidature for heart transplantation due to their condition of constant PAH were selected. Constantly severe PAH was defined as pulmonary artery systolic pressure (PASP) > 60 mmHg or pulmonary vascular resistance (PVR) > 3.0 Woods units, after a vasodilation test during right catheterization.^12^

The outcomes analyzed were the reduction of pulmonary artery pressures within 30 days of VAD implantation, analyzed by means of transthoracic echocardiogram, and one-year survival. This present study was approved by our institution's ethics committee on July 13, 2020 (#1799; CAAE 33484320.5.0000.5461).

## RESULTS

Out of the total of 23 patients who received VAD during the period from 2013 to 2019, four had had an indication for VAD implantation due to constantly severe PAH, as a bridge to candidature for heart transplantation. The devices implanted were Heart Mate II and Heart Mate III, with half of the sample in each group. The patients’ clinical characteristics are demonstrated in Table 1. Their mean age was 52 years, 50% were male, 75% had ischemic etiology and the mean ejection fraction of the left ventricle was 28%.

**Table 1:** Characteristics of the patients who underwent ventricular assistance devices (VAD) implantation

Characteristics		Number of patients = 4
**Average age (years)**		51 ± 13
**Male, n (%)**		2 (50)
**Ischemic cardiomyopathy, n (%)**		3 (75)
**INTERMACS 3, n (%)**		4 (100)
**Creatinine, mg/dl**		1.28 ± 0.26
**Sodium, mEq/l**		134.3 ± 4.1
**Hemoglobin, g/dl**		12.0 ± 2.8
**Albumin, g/dl**		3.8 ± 0.4
**Mean ejection fraction of LV, n (%)**		28 ± 1
**LV diastolic diameter (mm)**		69 ± 4
**PASP (mmHg)**		65 ± 4
**HeartMate mean risk score**		1.1 ± 0.8
**Implanted device, n (%)**		
	HeartMate II™	2 (50)
	HeartMate III™	2 (50)

Continuous variables expressed in ± standard deviation, SD; LV = left ventricular; PASP = pulmonary artery systolic pressure.

Table 2 presents the hemodynamic variables of the four patients before implantation of the VAD. These variables were obtained through right catheterization. All the patients were classified as INTERMACS 3, with a mean HeartMate II risk score of 1.1. The mean PASP in the preoperative period before VAD implantation in these patients was 65 mmHg ± 4 mmHg.

**Table 2:** Hemodynamic variables obtained by means of right cardiac catheterization prior to ventricular assistance device (VAD) implantation

	PASP (mmHg)	mPAP (mmHg)	SBP (mmHg)	mBP (mmHg)	PAOP (mmHg)	CO (l/min)	PVR (Wood units)	Post-PASP* (mmHg)	LVDD (pre)	LVDD (post)
Patient 1	67	43	87	53	40	5.1	0.6	33	67	67
Patient 2	60	32	84	68	23	1.6	5.6	51	75	65
Patient 3	64	38	80	53	22	5.2	3.1	31	66	66
Patient 4	70	48	100	80	20	2.6	10.8	28	68	62

* Value obtained by means of echocardiogram within 30 days after VAD implantation; hemodynamic measurements obtained after pulmonary reactivity test (patient 1: milrinone + dobutamine; patient 2: dobutamine + nitric oxide; patient 3: dobutamine + nitroprusside; patient 4: dobutamine + milrinone + nitric oxide); PASP = pulmonary artery systolic pressure; mPAP = mean pulmonary artery pressure; SBP = systolic blood pressure; mBP = mean blood pressure; PAOP = pulmonary artery occlusion pressure; CO = cardiac output; PVR: pulmonary vascular resistance; LVDD = left ventricular diastolic diameter.

**Figure 1:**
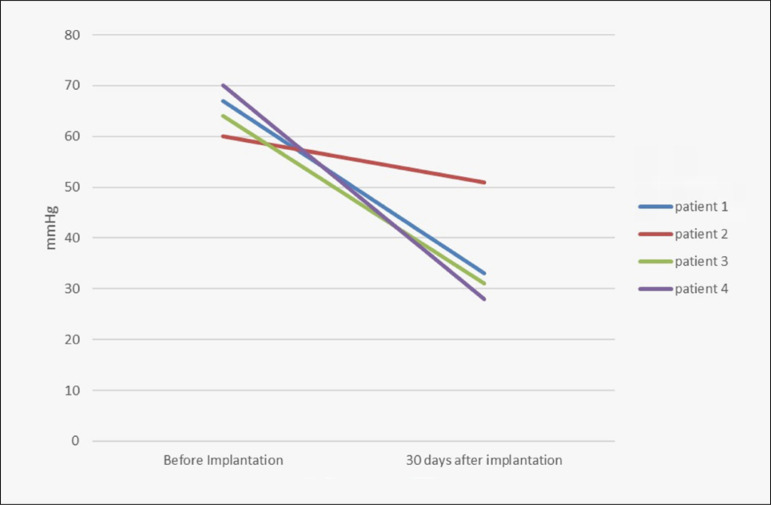
Pulmonary artery systolic pressure (PASP) levels in mmHg before ventricular assistance device (VAD) implantation (hemodynamic evaluation) and 30 days after VAD implantation (noninvasive echocardiogram evaluation).

Over the 30-day period after the procedure, all the patients evolved with a fall in PASP to below 60 mmHg, with an average value of 36 mmHg ± 10 mmHg (Figure 1). One of the patients underwent heart transplantation: this outcome can be ascribed to having achieved a reduction in PAH levels. The other patients continued to use the device, while presenting significant improvements in their functional class. None of the patients were using oral drugs for PAH management (such as phosphodiesterase-5 inhibitors). The one-year survival of this sample was 100%.

## DISCUSSION

This is the first Brazilian report on early effective reduction of constant PAH through long-term VAD use. This case series showed an average reduction of PASP over 30 days, from 65 mmHg ± 4 mmHg to 36 mmHg ± 10 mmHg (P = 0.256). The results show that decompression of the left ventricle by the VAD acted to reduce the pressure of left ventricle filling and that, retrogradely though the principle of communicating vessels, it acted to reduce pulmonary pressure, which is a determining factor for eligibility for heart transplantation.

Pulmonary hemodynamic evaluation remains an essential examination for inclusion of patients in the heart transplantation waiting list, through enabling evaluation of the degree of pulmonary hypertension and its reversibility with vasodilators. The main data obtained from this evaluation are the PASP, mean pulmonary artery pressure (mPAP), pulmonary artery occlusion pressure (PAOP) or pulmonary capillary pressure (PCP), transpulmonary gradient (TPG) and PVR.^4^ High levels of pre-transplantation pulmonary pressure, resistance and gradient have been correlated with increased post- heart transplantation mortality rates. These are therefore considered to be contraindications for this procedure, given the high risk of failure of the right ventricle of the graft.

Use of VAD as a tool capable of reducing PAH was first demonstrated in pulsatile flow devices.^5,6^ It is now known to also be efficacious with continuous flow devices.^3–9^ Zimpfer et al. demonstrated, in a cohort of 35 patients with a six-week follow-up, that there was a drop in mPAP levels by about 18 mmHg,^4^ which is compatible with the results demonstrated in our study. In another cohort of 50 patients, Ranjit et al found that there was a reduction in mean PASP of 20 mmHg, in an evaluation conducted three months after implantation of the device.^10^ These results allow us to infer that the reduction in PASP occurs mainly in the first 30 days and is maintained over the passage of the months.

We noticed that results similar to those described here had been found earlier, i.e. with favorable results within less than the 30-day period of our analysis, even without use of drugs to aid in this process, such as phosphodiesterase-5 inhibitors.

### Limitations

The main limitation that impeded provision of additional data from this observational study was the absence of pulmonary hemodynamic evaluation by means of a pulmonary artery catheter after VAD implantation. Evaluation of PASP was performed by means of echocardiography. However, the data obtained from this cohort of patients in Brazil allowed us to confirm the findings from studies conducted elsewhere in the world, thus emphasizing the importance of VAD devices as a bridge to candidature for heart transplantation, among patients for whom this is initially contraindicated due to constant PAH.

## CONCLUSION

Considering that all patients who underwent VAD implantation achieved early reduction of pulmonary pressures, thus enabling candidature for heart transplantation, this study opens up the possibility of new approaches with protocols for use of VAD as a bridge for candidature among patients with constant PAH, such that these patients can subsequently be offered heart transplantation.
